# Geographic access to emergency obstetric services: a model incorporating patient bypassing using data from Mozambique

**DOI:** 10.1136/bmjgh-2018-000772

**Published:** 2019-07-01

**Authors:** Emily B Keyes, Caleb Parker, Seth Zissette, Patricia E Bailey, Orvalho Augusto

**Affiliations:** 1 Reproductive, Maternal, Newborn and Child Health, FHI 360, Durham, North Carolina, USA; 2 Behavioral, Epidemiological and Clinical Sciences, FHI 360, Durham, North Carolina, USA; 3 Averting Maternal Death and Disability Program (AMDD), Heilbrunn Department of Population and Family Health, Mailman School of Public Health, Columbia University, New York, USA; 4 Universidade Eduardo Mondlane, Maputo, Mozambique

**Keywords:** geographic information systems, health systems, maternal health, obstetrics

## Abstract

**Introduction:**

Targeted approaches to further reduce maternal mortality require thorough understanding of the geographic barriers that women face when seeking care. Common measures of geographic access do not account for the time needed to reach services, despite substantial evidence that links proximity with greater use of facility services. Further, methods for measuring access often ignore the evidence that women frequently bypass close facilities based on perceptions of service quality. This paper aims to adapt existing approaches for measuring geographic access to better reflect women’s bypassing behaviour, using data from Mozambique.

**Methods:**

Using multiple data sources and modelling within a geographic information system, we calculated two segments of a patient’s time to care: (1) home to the first preferred facility, assuming a woman might travel longer to reach a facility she perceived to be of higher quality; and (2) referral between the first preferred facility and facilities providing the highest level of care (eg, surgery). Combined, these two segments are total travel time to highest care. We then modelled the impact of expanding services and emergency referral infrastructure.

**Results:**

The combination of upgrading geographically strategic facilities to provide the highest level of care and providing transportation to midlevel facilities modestly increased the percentage of the population with 2-hour access to the highest level of care (from 41% to 45%). The mean transfer time between facilities would be reduced by 39% (from 2.9 to 1.8 hours), and the mean total journey time by 18% (from 2.5 to 2.0 hours).

**Conclusion:**

This adapted methodology is an effective tool for health planners at all levels of the health system, particularly to identify areas of very poor access. The modelled changes indicate substantial improvements in access and identify populations outside timely access for whom more innovative interventions are needed.

**Key questions**

**What is already known?**

- Common measures of access do not consider a woman’s bypassing behaviour or the time it takes to reach services, relying instead on distance to services or ratios of facilities to population.

**What are the new findings?**

- Using facility characteristics known to influence women’s choice of facility and modelling within a geographic information system, we attempt to produce more accurate measures of access by accounting for trade-offs women make when deciding where to seek care.
- Common interventions—upgrading facilities to provide additional services and providing emergency transportation—improve access for a substantial proportion of the population; yet, some populations remain far outside timely care.

**What do the new findings imply?**

- Methodologies such as those used in this paper can provide actionable data for health planners to evaluate the impact of interventions.
- Common interventions to improve access are not sufficient to reach universal, timely access; areas with persistently poor access must be targeted with more innovative interventions to ensure equity.

## Introduction

The global maternal mortality ratio has declined by 44% since 1990, though these gains have been geographically disparate and many countries, including Mozambique, did not reach targets set by the Millennium Development Goals.^1^ To further reduce mortality to the ambitious targets set by the Sustainable Development Goals, recent gains must be expanded through targeted responses that consider not only national contexts but also the complexity of health system capability, and subnational and local contexts.^2 3^ Providing planners with better information on variations in geographic access to lifesaving services could help countries target appropriate strategies.^4^

One traditional measure of geographic access, the ratio of facilities per population, is a crude proxy of a population’s access to services. Yet, it remains a core measure at global and national levels.^5^ Facilities per capita ratios can mask geographic inequities; when measured against a target, a province may have sufficient facilities per population, but their geographic distribution could leave a substantial percentage of the province’s population unable to reach timely care. These ratios can be calculated at lower administrative levels, but targets for adequate coverage of emergency services are difficult to interpret for small populations,^5^ rendering them inadequate measures for local health planning. Furthermore, facility-to-population ratios do not account for the time needed to reach services, despite substantial evidence that links improved health outcomes with time to health services.^6–8^

Understanding access beyond facility-to-population ratios requires estimates of distance or travel time to services. The use of geographic information system (GIS) allows one to determine distance and time, either through simple estimation of straight-line distances between two points (ie, Euclidean distance)^9 10^ or through distance along walking paths and road networks.^11 12^ Yet, distance measures alone do not consider the impact of additional geographic barriers on travel time to services and can generate misinformed estimates of access.^12^ Travel time measures can account for variations in rates of travel across varying slopes, ground cover and road surfaces as well as availability of emergency transportation. The application of GIS in maternal and newborn health is growing, as is the sophistication of access modelling.^13 14^ Some models have validated the predictive accuracy of spatial accessibility^12 15^; accounted for the availability of public transportation,^16^ referral between facilities, and the impact of modifications to the distribution of services and infrastructure^11^; and applied sophisticated models that define catchment areas.^17 18^

Some of the aforementioned approaches are limited by the assumption that a woman selects the closest health facility offering the level of service she needs.^9–11 16^ Evidence suggests that a woman’s choice of facility and whether she uses or bypasses the nearest facility is influenced by individual and facility-level characteristics related to perceptions about quality of services.^19–23^ Evidence indicates that the odds of a facility being bypassed increase if women perceive it to provide poor quality care and decrease if quality is perceived to be high. Availability of drugs and equipment, cleanliness, privacy of the delivery room and health provider attitudes influence women’s perceptions of quality,^20–22^ as does the number of emergency obstetric care (EmOC) signal functions provided at the facility.^20 22 23^ A study in Tanzania found that for every additional signal function provided the odds of that facility being bypassed were almost halved.^23^ When they do bypass, women appear satisfied with their choice. Compared with non-bypassing mothers, bypassing mothers have reported receiving better care, better availability of drugs and equipment, and higher levels of satisfaction with delivery services, despite having paid more and travelled further for services.^22^

The objective of this paper was twofold. First, we aimed to adapt the existing methodology modelling geographic access to better reflect the complexity of women’s behaviour using facility characteristics known to influence women’s care-seeking choices. Second, we aimed to demonstrate the utility of the adapted methodology by applying it to measure the impact of common improvements on efforts to achieve universal timely access to emergency care, using data from Mozambique.

## Methods

Mozambique, a low-income country in southern East Africa, had a gross national per capita income of US$480 in 2016.^24^ Divided into 11 administrative units (provinces), including the capital city of Maputo, the country is crossed from west to east by large rivers, isolating some parts during the rainy season. The population in 2012 was 23.6 million.^25^ The national health system provides the vast majority of healthcare services, while private sector facilities are available primarily in urban areas.^26^ The health system has four levels of progressively complex care: the first level (health centres and posts) provides primary care including basic maternal and child health services; the secondary level (rural, district or general hospitals) may offer surgical services and serves as a referral level for the first; the tertiary level (provincial hospitals located primarily in capitals) functions as the next referral level; and the quaternary level (central hospitals) serves as the regional referral level.^26^ However, women’s choice of facility does not necessarily follow this clearly defined pyramidal structure, particularly in emergencies. The maternal mortality ratio in Mozambique has remained high, hovering around 480/100 000 live births, over the past two decades.^27^ In 2012, the proportion of deliveries occurring in facilities was 67%, yet the caesarean delivery rate was 2.8% of all expected deliveries, indicating that not all women needing this life-saving intervention are able to access it.^28^

Using multiple data sources and modelling within a GIS environment, we calculated two segments of a patient’s journey to care. The first segment was home to the first preferred facility assuming women, if given a choice, would travel longer to reach a facility perceived to be of higher quality. If the first preferred facility did not offer the highest level of service, the woman may need to be referred upwards to the closest facility that did. This would be the woman’s second journey segment. Together these two segments made up the total travel time to highest care. We then used these models to measure resulting changes in the proportion of the population with access to the highest level of care after upgrading strategically located facilities to provide higher-level services and placing ambulances and communication modes at midlevel to lower-level facilities.

### Facility data

Facility data from Mozambique’s 2012 assessment of emergency obstetric and newborn care were used with permission from Mozambique’s Ministry of Health. Data were collected between November and December 2012 through a cross-sectional survey of health facilities. The survey included a census of all health facilities that provided delivery services in the previous year, regardless of volume (946 facilities). Six data collection modules were used in the assessment, though this secondary analysis included data from just four^28^: facility infrastructure; human resources; essential drugs, equipment and supplies; and facility case statistics. Data collectors were medical students in their final year or recent medical graduates. All were trained with a standardised curriculum over 5 days. The survey received approval from the local ethics committee. Data were double-entered into EpiData and exported to Stata V.13.^29^ For complete methods, see the final survey report^28^; for distribution of facilities by type and province, see online supplementary file 1, Table 1.1.

10.1136/bmjgh-2018-000772.supp1 Supplementary file 1

A master list of facility geographic coordinates was provided by the Ministry of Health. This list, accurate as of 2012, included 1266 health facilities, some of which did not provide delivery services. Using data management techniques in Excel and multiple rounds of manual matching, we matched health facilities in the assessment to facilities on the master list. For those not on the master list, we triangulated data from facility location information in the assessment (eg, facility name, district, zone, region) with data available through several online sources (Google Maps, Google Earth, GeoNames, OpenStreetMap and The Fuzzy Gazeteer) to identify a settlement that matched the facility location. We then selected coordinates from a central location of that settlement. If a settlement was large, more than one settlement was possible, or we found no likely settlement, that facility was excluded from our analysis (online supplementary file 1, Table 1.2).

### Defining levels of care

For each facility, we used assessment data to calculate a score based on facility characteristics known to influence women’s *perceptions* of quality. We did not attempt to categorise facilities by actual quality of service. We also did not use the formal definition of fully functioning basic EmOC and comprehensive EmOC, as determined by *recent performance* of the life-saving interventions known as the EmOC signal functions.^5^ Rather, our score was graduated along five levels that were largely based on the facility’s *readiness* to provide each of the nine signal functions (online supplementary file 2, Table 2.1). Using a facility’s signal function readiness rather than actual provision allowed inclusion of facilities that might not have provided the function recently, perhaps due to low patient volume, but theoretically could have if a patient needed it. The score had a maximum of 14 points and was based on two dimensions:

- *General readiness (five points maximum)*. Facilities received one point for each of five items present: open all day every day, availability of electricity, availability of water, any type of functioning transport and a functioning mode of communication.
- *Signal function readiness (nine points maximum)*. Facility readiness to provide each signal function was determined by the presence of staff who could provide the intervention and the minimum drugs, equipment and supplies required to do so. The facility earned one point for each signal function for which it was staffed and equipped. Human resource availability was determined by asking whether a health worker was available who could provide each function. If so, the facility was considered minimally staffed for that function. Categories of health workers were obstetrician, paediatrician, general doctor/physician, four levels of midwife, surgical technician and medical technician.^28^ Anaesthetists and anaesthesiologists were not asked about in the data collection instrument; however, the ability to provide general or regional anaesthesia was asked of each category of worker, and was a requirement for caesarean readiness. Facilities were determined to be equipped to provide the signal function if they had the minimum required combination of drugs, equipment and supplies in stock on the day of the survey (online supplementary file 2, Table 2.1).

10.1136/bmjgh-2018-000772.supp2 Supplementary file 2

Based on the facility score and two other items—presence of functioning transport and recent performance of caesarean delivery—we ultimately placed facilities in one of five levels (table 1) (detail in online supplementary File 2 Section 2.2).

**Table 1:** Definition of facility levels and time-bounded catchment areas

Facility level	Readiness score	Other criteria	Catchment areas (prioritisation)	
			Primary catchment	Secondary catchment
5	10–14	Performed caesarean delivery in the last three months	0–2 hours (first)	2–5 hours (fifth)
4	10–14		0–2 hours (second)	2–5 hours (sixth)
3	0–9	Has functional motorised transport	0–1 hour (third)	1–3 hours (seventh)
2	6–9		0–1 hour (fourth)	1–3 hours (eighth)
1	0–5		Not applicable	0–1 hour (ninth)

### Geographic layers

The access models were created in stages within ArcGIS V.10.3 software^30^ using the Spatial Analyst and Network Analyst extensions. To calculate travel time to health facilities, a single cost–distance raster layer estimated the cost in minutes required to cross each cell. The cost was determined using three data sources: land cover, road networks and elevation. Land cover^31^ was used to represent likely pedestrian travel times, and walking speeds were defined for each type of land cover.^32^ The road network vector data set was downloaded from OpenStreetMap^33^ and used to estimate motorised travel speeds.^34^ We replaced pedestrian values with motorised travel speeds in land cover cells where roads overlapped; some road cells overlapped empty spaces once occupied by river features, indicating bridges that pedestrians and vehicles could move across. Finally, this raster of combined pedestrian and motorised rates was attenuated by slope using a digital elevation model^35^ and the Van Wagtendonk formula.^36^ Additional information, including assumed rates of travel, can be found in online supplementary File 3. The population data set used was created by WorldPop with a population projection to the year 2010, in a raster format with approximately 100 m × 100 m cells.^37^ The 2010 population was used in the modelling to establish proportions of the population with various levels of access. Absolute values of population were calculated based on population projected to 2012 to align with the year of the Mozambique facility assessment. Where estimates of expected pregnancies and severe obstetric complications are reported, they were determined by applying Mozambique’s 2012 crude birth rate to the 2012 population^25^ and the estimate that 15% of expected pregnancies will result in severe complications.^5^

10.1136/bmjgh-2018-000772.supp3 Supplementary file 3

### Modelling

We defined catchment areas with time-bound limits to simulate decision points at which women might evaluate a trade-off between perceived quality of services and time to care (table 1). The health system must be organised to ensure timely, universal access for all scenarios pregnant women may face. We framed our model assumptions for measuring timely access against the most time-critical complication, postpartum haemorrhage, thus the selected time bounds include the clinically relevant 2-hour mark and a maximum travel time of 5 hours.^38^ These time-bound intervals were exploratory, as we found no literature that investigated the travel time parameters around women’s choice of facility. Women’s decision points are likely more fluid; yet, to operationalise the approach we defined finite ranges. Each time-bound catchment area was prioritised beginning with primary catchment area level 5, followed by level 4, and then lower levels (table 1).

#### Journey segment 1: home to first preferred facility

**Figure 1:**
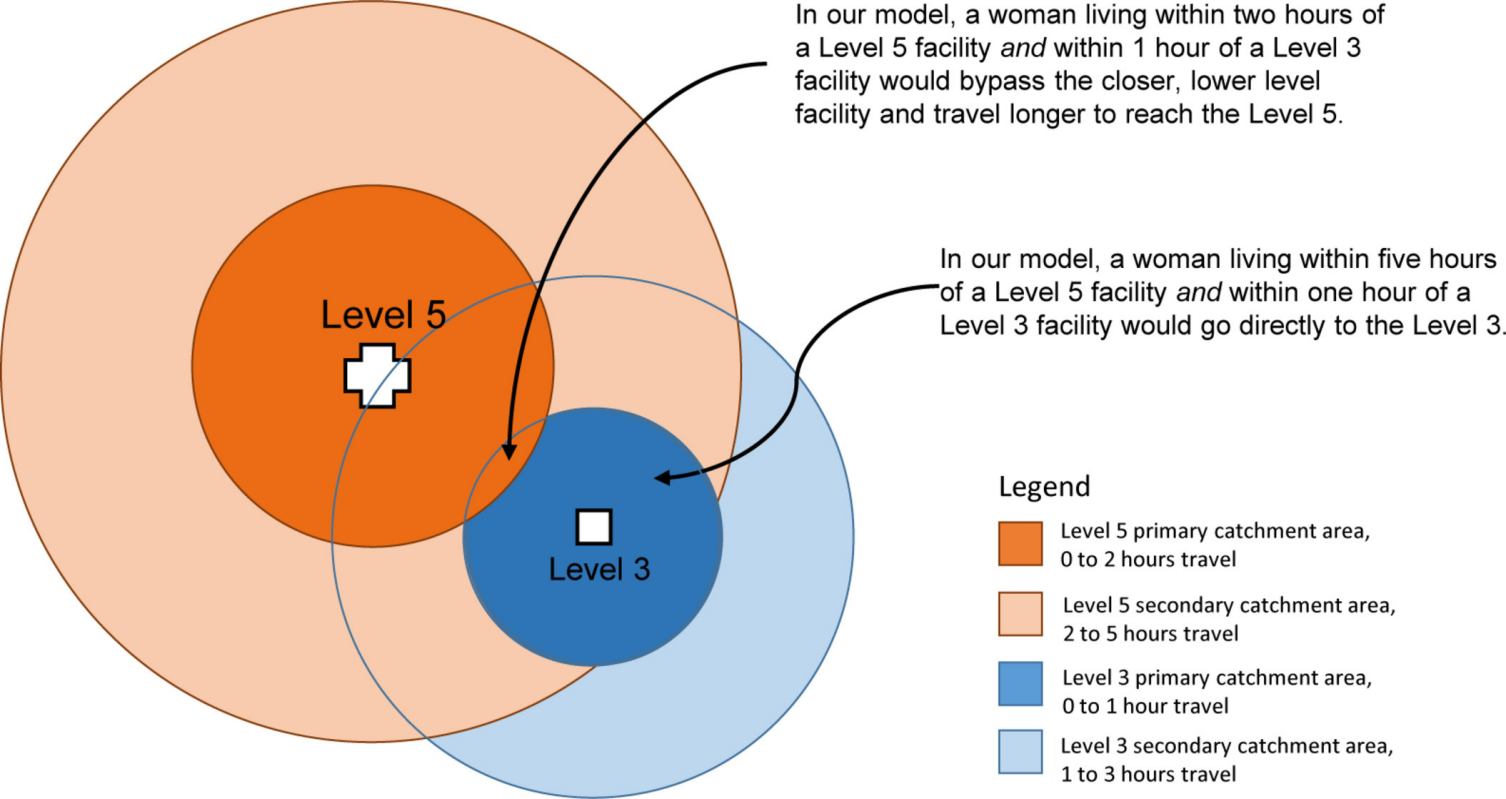
Illustrative diagram of 1-hour catchment area boundaries around two facilities, their relative priority and resulting modelled behaviour.

In ArcGIS, we layered health facilities onto the cost–distance raster described above, and for each facility created polygons of time-bound catchment areas outwards into the surrounding space until the primary and secondary travel time thresholds were reached. This generated multiple overlapping time-bound catchment areas—one for each facility’s primary and secondary catchment areas. For cells where catchment areas overlapped, we maintained the area with the highest priority and deleted the others. This created a complex patchwork of catchment areas modelling how women in these areas might bypass a nearby facility in favour of one further away. Figure 1 helps visualise this approach: if a woman lives within the primary catchment area of a level 3 (defined by 0–1 hour of travel time) and within the primary catchment area of a level 5 (defined as between 0 and 2 hours of travel time), our model prioritises the level 5 catchment area and assumes she would choose to bypass the closer level 3 facility in favour of accessing the perceived better-quality services of the level 5 facility. In this article, that level 5 would be her *first preferred facility*. Alternately, if a woman lives within the primary catchment area of the level 3 and within the secondary catchment area of the level 5, the model assumes she would travel directly to the level 3, and that would be her *first preferred facility*.

Finally, we overlaid the population layer to calculate the population with access to their first preferred facilities within hourly segments.

#### Journey segment 2: interfacility referral

For women whose first preferred facility is not a level 5, we modelled interfacility referral using the ArcGIS Network Analyst extension to calculate the direct transfer time between each facility and the closest level 5. Transfer time was estimated along the road network based on assumed travel speeds for each road type. We adjusted the direct transfer time per the availability of vehicles and communication at the sending facility: if the facility had a functioning vehicle, the total transfer time was maintained as the direct time; however, if the facility had no vehicle, the transfer time was doubled since we assumed the receiving facility would send a vehicle to retrieve the patient. All but one level 5 had transport; our status quo model would require functional transportation be placed there. If the sending facility did not have communication, we further increased the transfer time by 30 min to allow time to locate a phone.^11^ To describe changes in journey segment 2, we reported the population-weighted interfacility transfer times. It was necessary to weight by population because the units of analysis were segments of catchment areas of varying population sizes.

#### Total travel time

In Stata V.13, we calculated total travel time to a level 5 by adding travel times of both journey segments. To evaluate changes across models, we reported population-weighted total travel times.

We then applied these methods to four models:

- Model 0: This is the current situation of access, status quo.
- Model 1: We identified 37 level 4 facilities that were each suitable for upgrade to level 5, based on a high facility score and degree of geographically strategic location.
- Model 2: We located vehicles at the 659 facilities without transportation and phones at the 140 facilities without communication.
- Model 3: We combined the improvements of models 1 and 2.

### Patient involvement

No patients were involved in this research.

## Results

### Facility characteristics

Eighty facilities (9%) were excluded from the analysis because no geographic coordinates were found. In all but four regions (Sofala, Manica, Nampula, Inhambane), >90% of surveyed facilities (delivery sites) were included in our models. Of note, 15 facilities categorised as level 4 were excluded from the models; including them would likely have improved modelled access to care, particularly for Gaza and Zambézia (online supplementary File 1, Table 1.2).

The distribution of key facility characteristics by facility level (table 2) supports the approach. All level 5 facilities were hospitals, as were 4% of level 4 facilities and 1% of level 3 facilities. The highest percentage of private facilities appeared in level 5 (21%). The mean number of monthly deliveries and the mean number of obstetric beds decreased from level 5 to level 1. Most level 5 facilities had a functional mode of motorised transportation (one did not), and by definition, all level 3 facilities did. Level 5 and 4 facilities were most likely to have a mode of functional communication on site, followed by level 2 facilities; communication was available in more than two-thirds of level 3 and level 1 facilities. The relative distribution of facilities, deliveries and complications by facility level further supports this classification (online supplementary File 4, online supplementary Figure 1).

10.1136/bmjgh-2018-000772.supp4 Supplementary file 4

10.1136/bmjgh-2018-000772.supp5 Supplementary file 5

**Table 2:** Per cent distribution of facilities according to facility type and sector, mean number of deliveries and obstetric beds, and per cent with critical referral infrastructure, by facility level, model 0

	All facilities (n=866)	Level 5 (n=43)	Level 4 (n=259)	Level 3 (n=83)	Level 2 (n=459)	Level 1 (n=103)
Facility type						
Hospitals	5.8	100.0	4.3	1.2	0.0	0.0
Health centres	94.2	0.0	95.8	98.8	100.0	100.0
Sector						
Public	91.4	79.1	87.9	92.8	93.4	95.2
Private	8.6	20.9	12.1	7.2	6.6	4.8
Number of monthly deliveries (mean)	57	300	72	54	35	18
Number of obstetric beds (mean)	6.8	30.4	9.0	5.4	4.3	4.0
Per cent with any functional mode of motorised transportation	30.0	97.7	62.2	100.0	0.0	0.0
Per cent with functional communication	85.2	97.7	95.8	69.9	85.2	66.0
Mean readiness score (out of 14)	8.3	12.5	10.9	8.1	7.6	3.6

### Modelling

Table 3 presents key results for each journey segment for status quo (model 0) and each improvement scenario (models 1–3). For journey segment 1, we present the percentage of the population able to reach any facility within the clinically critical 2-hour period, and the level of care that would be reached. We present the mean journey times for journey segment 2 and total journey, along with the 95th percentile threshold and the maximum times. Twenty-one per cent of the population does not live within a defined primary or secondary catchment area and thus remained outside our models.

**Table 3:** Key results for each journey segment

		Model 0 Status quo		Improvement scenarios					
				Model 1 Upgrading 37 facilities to function as level 5		Model 2 Extending referral infrastructure to all lower-level facilities		Model 3: combined Upgrading 37 facilities and referral infrastructure to all facilities	
		2012 population	%	2012 population	%	2012 population	%	2012 population	**%**
*Journey segment 1*									
Within 2 hours, can directly reach									
Level 5		9 656 182	41.0	10 625 628	45.1	9 656 185	41.0	10 625 628	45.1
Level 4		2 777 829	11.8	1 808 385	7.7	2 777 830	11.8	1 808 385	7.7
Level 3		25 931	0.1	26 404	0.1	234 961	1.0	257 102	1.1
Level 2		183 177	0.8	183 177	0.8	–	–	–	–
Level 1			–	–	–	–	–	–	–
No facility reached within 2 hours		10 926 789	46.4	10 926 314	46.4	10 900 932	46.2	10 878 793	46.2
Total		23 569 908	100.0	23 569 908	100.0	23 569 908	100.0	23 596 908	100.0
*Journey segment 2*									
Among those needing transfer to reach a level 5, population-weighted travel time (in hours)	Mean (SD)	2.9 (2.1)		2.7 (2.1)		2.0 (1.2)		1.8 (1.1)	
	95th percentile	8.4		8.5		4.0		4.0	
	Max	16.8		13.1		8.4		6.3	
*Total journey*									
Population-weighted travel time (in hours)	Mean (SD)	2.5 (2.2)		2.2 (1.9)		2.3 (1.8)		2.0 (1.6)	
	95th percentile	6.8		5.4		5.8		5.1	
	Max	19.3		15.6		12.9		9.3	

#### Model 0: status quo

In model 0, 41% of the population could reach a level 5 within 2 hours, and an additional 12% of the population could reach a level 4 within this time frame. Just more than half of the country’s population could reach their first preferred facility within 2 hours, leaving 46% (10.9 million people representing 414 200 expected pregnancies and 62 130 expected severe complications) still *en route* after 2 hours. All level 1 facilities would be bypassed in our models. In model 0, among those individuals needing to be referred upwards to level 5, the population-weighted mean transfer time was 2.9 hours. For total journey time, the mean was 2.5 hours, and the maximum was 19.3 hours. The 95th percentile threshold indicates that 5% of the included population had a total journey time more than 6.8 hours. Adding the 21% of the population outside the models, around one-quarter of the population likely has a total journey of >6.8 hours.

Mean segment 2 journey time was longer than the mean total journey time because it was calculated on a subset of the population—those whose first preferred facility is not a level 5 and who therefore need to be transferred upwards. In contrast, the mean total journey time was calculated among the full model population, many of whom would arrive directly at a level 5 and would not have a second journey segment.

#### Model 1: upgrading facilities

Model 1 estimated the impact of assigning a level 5 classification to 37 geographically strategic level 4 facilities, nearly doubling the number from 43 to 80. The results indicated that an additional 4% of the population (or an additional 968 846 people; 36 916 pregnancies; 5522 severe complications) would reach a level 5 directly within 2 hours. Journey segment 2 would be reduced by 12.6 min on average, from 2.9 to 2.7 hours. The mean total journey time would be reduced from 2.5 to 2.2 hours, and the 95th percentile threshold decreased by 1.4 hours.

#### Model 2: locating ambulances/transportation and communication where none exist

Model 2 estimates the impact, on journey segment 2 and the total journey, of providing modes of transport and communication to lower-level facilities (levels 1–3). The distribution of the population for journey segment 1 was essentially unchanged from model 0 because this improvement would only affect journey segment 2. However, the mean transfer time was reduced by almost one-third between models 0 and 2—from 2.9 to 2.0 hours, a 52 min reduction. The mean total journey was only modestly reduced; however, the 95th percentile threshold decreased by 1.0 hour, and the maximum transfer time was reduced by half.

#### Model 3: combination of the two improvements

The combination of improvements yields gains in both journey segments and overall. The distribution of the population by level of first preferred facility remained similar to model 1, yet the mean segment 2 time was reduced by just more than 1 hour, to 1.8 hours. The mean total journey time was reduced from 2.5 hours in model 0 to 2.0 hours in model 3. Furthermore, there was improvement for women in the most remote areas, with the maximum total travel time more than halved, and the 95th percentile threshold reduced from 6.8 to 5.1 hours.

We also looked at women’s movement in and out of facilities across time and compared this movement between models 0 and 3 (online supplementary File 4).

### Geographic distribution of improvements in access

One important utility of modelling access within a GIS is the ability to specify geographic areas that would benefit from modelled improvements, as well as areas that would remain underserved.

**Figure 2:**
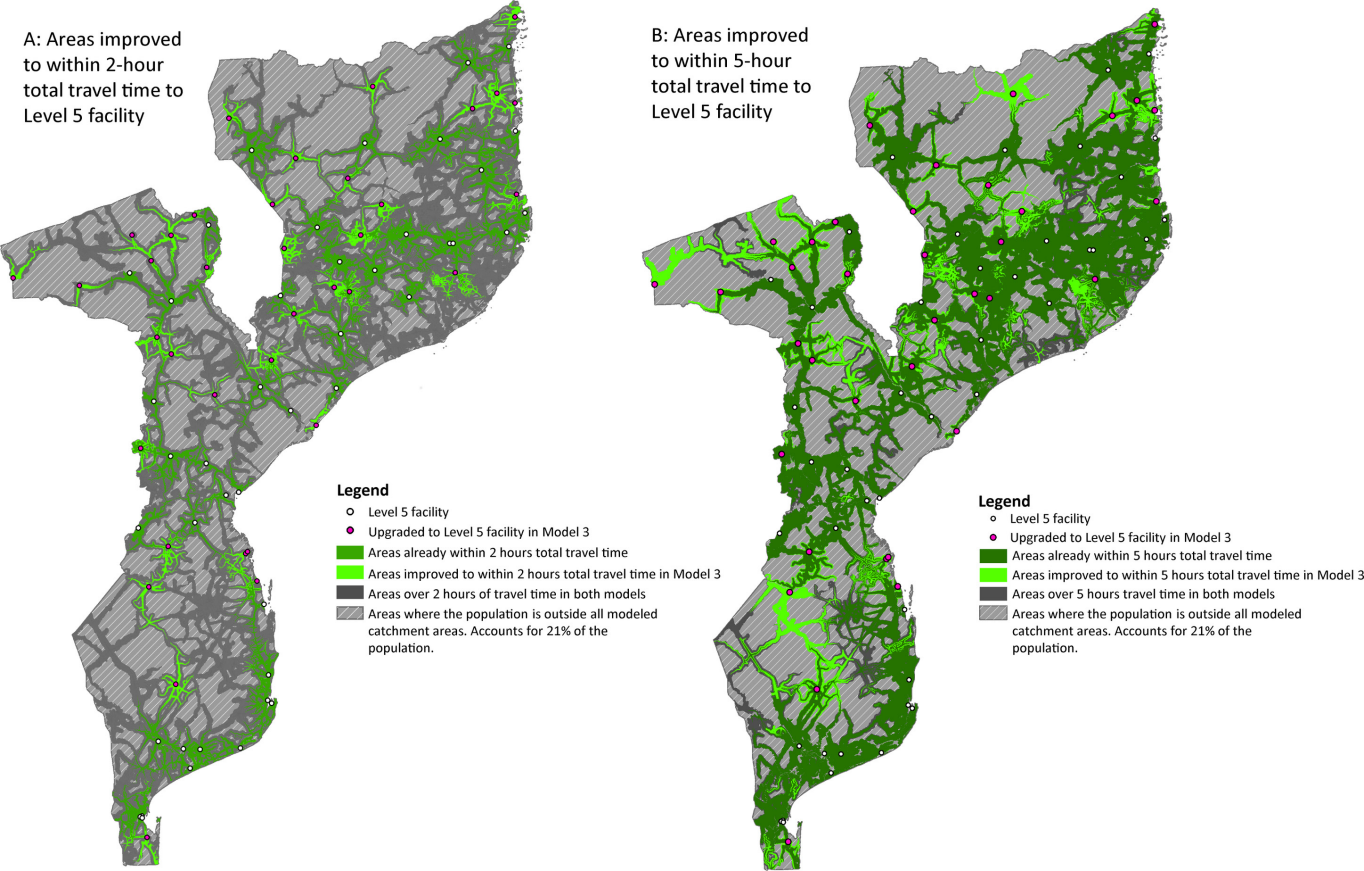
Geographic distribution of improved 2-hour and 5-hour access to level 5: model 0 versus model 3.

Figure 2 shows the precise geographic areas (bright green) where model 3 improvements changed access to a level 5 from >2 hours to <2 hours (A), and from >5 hours to <5 hours (B). All areas of improvement appeared around one of the 37 upgraded level 5 facilities (shown in pink), indicating the extent to which that approach fills gaps in timely access. Looking at the maps in conjunction with online supplementary File 4 and online esupplementary figure 2, one could quantify the population that lives in the bright green areas. Model 3 increased the population with 2-hour access to a level 5 by 4 percentage points (42% reaching a level 5, whether directly or via referral in model 0, compared with 46% in model 3). The maps also indicate areas (dark grey) where access, though improved, would remain inadequate (>2 hours, or >5 hours, to a level 5). Finally, geographic areas where baseline access is poor are indicated (grey stripes). This area represents 21% of the population, described as outside the models, that would remain *en route* after 5 hours in each of our models.

10.1136/bmjgh-2018-000772.supp6 Supplementary file 6

## Discussion

This paper’s first aim was to demonstrate a refined methodology to estimate women’s access to emergency obstetric services incorporating evidence that choice of facility, though influenced by time to care, is also influenced by perceived quality of services. This approach is a fundamental improvement on others that assume women seek care at their closest facility. The approach also leverages the evidence that the number of EmOC signal functions provided, along with other basic facility characteristics, are strong predictors of facility choice.^20 22 23^ Our second aim was to apply this methodology to model scenarios of common strategies for improving emergency access in Mozambique to estimate the impact on access over the status quo. The models’ results describe how close Mozambique’s health system is to ensuring universal, timely access to emergency services.

Doubling the number of facilities providing the highest level of service and making substantial investments in transportation and communication would increase the population with 5-hour access to the highest level by almost 1.5 million people (57 000 expected pregnancies and 8550 expected severe complications), and increase access within 2 hours by almost 950 000 people (36 100 expected pregnancies and 5415 expected severe complications). Furthermore, the mean time it would take a woman to move between her first preferred facility and the highest level of care would be reduced by just over 1 hour. Despite these substantial gains, almost one-third of the population, and a relative proportion of the expected pregnancies and complications, would remain beyond 5-hour access to the highest level of care, and the mean total journey time would remain >2 hours across all models. Neither the status quo nor the envisioned improvements are sufficient to ensure universal timely access.

Some model limitations make our estimates of access conservative. By design, our models capture the population with access to a lower-level facility within 1 hour, or to a higher-level facility within 3 or 5 hours. Twenty-one per cent of the population not in this category was excluded from the models. Women in this population have very poor access, yet some would reach a lower-level facility at some point within 5 hours, though not a level 5; thus, the population *en route* after 2 hours has been overestimated. Roads increase access. For areas where roads exist but are not found in OpenStreetMap data, our models may underestimate access although we feel confident that the augmented road network data used is quite complete. Also, 80 facilities that could not be geographically located were excluded. In all models, a very small proportion of women access a level 1, 2 or 3 facility as first preferred facility. Therefore, the exclusion of the 65 level 3 or lower facilities is unlikely to have substantially affected our results. However, had the 15 level 4 facilities been included, access in all models would have been improved, particularly in Gaza and Zambézia provinces.

On the other hand, these models in many ways represent optimal conditions, and real access would likely be poorer. For example, the models estimate travel during the dry season and daytime, when roads are most traversable. Other model assumptions could be used to estimate travel times under less favourable conditions; however, seasonal variation fluctuates even within the wet and dry seasons, and complex models are required to understand this well.^39^ The travel times represent only the physical movement across space; they do not account for delays in the decision to seek care, waiting for transportation once reaching a road, time to be seen by a doctor or delay in a provider’s decision to refer. A woman’s true time to care, therefore, would be longer. Little evidence exists on the magnitude of these delays; thus, we did not include them.

Some important factors and methodological approaches were not used. Whether a facility charges for services, and how much, could impact a woman’s choice of facility. We did not include this characteristic in our model because >98% of facilities in Mozambique did not charge for routine or emergency obstetric services.^28^ Furthermore, the private sector in Mozambique serves a very small proportion of pregnant women, predominantly the wealthy and urban.^40^ In countries with a more robust private sector, and where poor women seek care despite the costs, the algorithm for classifying facilities should consider the impact of cost on choice. Our methodology could be improved by conducting sensitivity analyses on the parameters used in the models, as well as ground-truthing results. Funds and resources for ground-truthing were not available for our activity, though we are confident that the results remain useful for decision-makers. We used a geographic layer representing total population rather than pregnancies or women of reproductive age. Raster data of pregnancies and women of reproductive age are available, and they are derived from population data by making assumptions about sex and age distribution along with estimates of fertility. Some evidence indicates that the geographic pregnancy data in Mozambique require further adjustment to adequately represent reality; given this uncertainty, we used the population data set.^41^ Targeted approaches needing to understand the magnitude of women at risk of obstetric complication would benefit from using accurate geographic pregnancy data, and modelling that links geographic location of pregnancies with health facilities is critical for planning and for identifying hard-to-reach communities.^42^ Despite these limitations and caveats, the described approach provides a more realistic description of the health system and the way women may use it, thus providing useful information for efforts to ensure universal access.

Some efforts towards universal access have placed delivery services at the first level of the health system, sometimes via training of community health workers.^23 43^ Now, some have called for gradually shifting the provision of obstetric care away from these lowest levels, towards health centres and hospitals,^22^ in part to align with women’s apparent preferences to deliver in higher-level facilities.^23 44^ If quality delivery services are concentrated in upper-level facilities, whether by design or default, performance of the emergency referral system becomes increasingly critical. Attempts to shorten interfacility transfer time are paramount to reducing mortality, particularly among women in remote, hard-to-reach areas. Modelling, as demonstrated, could inform a rational reorganisation of the health and emergency referral systems, as well as inform attempts at equitable distribution of services. This analysis indicates that a combination of upgrading facilities and widely distributing transportation can substantially reduce the mean transfer time between facilities. It also indicates that those interventions alone are insufficient to ensure universal timely access. If universal access is a goal, geographic areas of persistently poor access must be targeted with other interventions, such as maternity waiting homes, telemedicine and community midwifery, along with task-sharing treatment of the most time-critical and lethal complications (eg, postpartum haemorrhage and newborn resuscitation).^45^ Additionally, intersectoral collaboration with road authorities would be critical, as strategic improvements in road surfaces would likely yield the most substantial improvements in access for women currently in hard-to-reach areas.

## Conclusion

The models represent more accurate estimates of current geographic access and the impact that specific structural improvements at the health facility level may have on improving that access. Supporting decision-makers with dynamic models like these can provide more realistic expectations of the impact that planned interventions may have and make the case for further innovation and complementary programming to ensure equitable access.
