# Supplementary material for: Geographic access to emergency obstetric services: a model incorporating patient bypassing using data from Mozambique
Source: BMJ Glob Health. 2019 Jul 1;4(Suppl 5):e000772. doi: 10.1136/bmjgh-2018-000772 (PMC6606078; doi:10.1136/bmjgh-2018-000772)
Supplement: Supplementary file 1 [file bmjgh-2018-000772supp001.pdf]

Supplementary File 1 for Geographic access to emergency obstetric services: a model incorporating patient bypassing using data from Mozambique

Supplementary Table 1.1. Regional populations and number of facilities included in assessment, by type and region

Province	Population (2012)[1]	Institutional delivery rate (2012)[2]	Total facilities in assessment	Facility Type: Central hospitals	Facility Type: Provincial hospitals	Facility Type: General, rural, and district hospitals	Facility Type: Health centers
National	23,569,908	72%	946	3	7	46	891
Niassa	1,443,460	80%	77	0	1	2	74
Cabo Delgado	1,797,335	84%	101	0	1	4	96
Nampula	4,620,399	66%	119	1	0	6	112
Zambézia	4,413,706	71%	163	0	1	9	153
Tete	2,228,527	59%	70	0	1	2	67
Manica	1,735,351	77%	78	0	1	4	73
Sofala	1,894,763	95%	81	1	0	6	74
Inhambane	1,426,684	79%	104	0	1	4	99
Gaza	1,342,045	83%	92	0	1	4	87
Maputo Provincia	1,473,516	27%	49	0	0	1	48
Maputo Cidade	1,194,121	89%	12	1	0	3	8

Sources: (1) National Statistical Institute (INE) 2011 Census, projected to 2012 (2% annual rate of growth); (2) Avaliação das Necessidades de Serviços de Cuidados Obstétricos e Neonatais de Emergência em Moçambique, 2012.

Supplementary Table 1.2 Number of facilities included and excluded from modeling, by province and facility level

	Total facilities in modeling		Level 1		Level 2		Level 3		Level 4		Level 5	
**Province**	excluded	included	excluded	included	excluded	included	excluded	included	excluded	included	excluded	included
National	80	866	18	85	40	418	7	76	15	244	0	43
Niassa	5	72	3	10	2	43	0	2	0	14	0	3
Cabo Delgado	10	91	2	4	7	43	0	8	1	32	0	4
Nampula	13	106	2	10	6	46	4	16	1	28	0	6
Zambézia	7	156	1	17	3	91	0	9	3	33	0	6
Tete	3	67	1	4	0	39	1	3	1	18	0	3
Manica	10	68	7	11	1	25	1	13	1	16	0	3
Sofala	11	70	1	7	7	20	1	9	2	28	0	6
Inhambane	11	93	1	7	10	51	0	4	0	26	0	5
Gaza	6	86	0	11	2	38	0	8	4	25	0	4
Maputo City	4	45	0	3	2	19	0	4	2	19	0	0
Maputo Province	0	12	0	1	0	3	0	0	0	5	0	3
