# Supplementary material for: Geographic access to emergency obstetric services: a model incorporating patient bypassing using data from Mozambique
Source: BMJ Glob Health. 2019 Jul 1;4(Suppl 5):e000772. doi: 10.1136/bmjgh-2018-000772 (PMC6606078; doi:10.1136/bmjgh-2018-000772)
Supplement: Supplementary file 2 [file bmjgh-2018-000772supp002.pdf]

Supplementary File 2 for Geographic access to emergency obstetric services: a model incorporating patient bypassing using data from Mozambique

2.1 Facility scoring

Supplementary Table 2.1. Drugs, equipment, and supplies required to provide each EmOC signal function

EmOC signal function	Minimum drugs, equipment, and supplies in stock
Parenteral antibiotics	Any intravenous or intramuscular antibiotic: gentamicin, ampicillin, metronidazole, or penicillin G sodium
Parenteral oxytocics	Oxytocin (injection)
Parenteral anticonvulsants	Magnesium sulfate
Manual removal of placenta	Sterile gloves
Removal of retained products of conception by manual or electric vacuum aspiration or by dilatation and curettage	Functioning electric vacuum aspirator or syringe for manual vacuum aspiration and various sizes of flexible cannulae, or at least one type of blunt curette and uterine dilators, and local anesthesia (lidocaine-1%, injection)
Assisted vaginal delivery by vacuum extraction	Functioning vacuum extractor with different sizes of cups
Resuscitation of newborn with bag and mask	Functioning Ambu bag, and mucus extractor or suction aspirator for newborn resuscitation
Blood transfusion	Microscope; reagents for blood typing and cross matching; empty blood bags; functioning refrigerator for blood bank; blood tests for HIV, hepatitis B, and syphilis
Obstetric surgery/cesarean delivery	Functioning oxygen cylinders (i.e., oxygen cylinders with manometer and flowmeter, or low-flow, tubes and connectors), operating table, general/regional anesthesia equipment (i.e., functioning anesthesia machine, masks for anesthesia, halothane or ketamine) or local anesthesia (i.e., lidocaine or bupivacaine) to be used as regional anesthesia

Sources: World Health Organization, USAID. Measuring Service Availability and Readiness: A Health Facility Assessment Methodology for Monitoring Health System Strengthening: Service Readiness Indicators (Geneva: WHO, 2012); World Health Organization. Monitoring the Building Blocks of Health Systems: A Handbook of Indicators and Their Measurement Strategies (Geneva: WHO, 2010); MEASURE Evaluation. Guidance for Selecting and Using Core Indicators for Cross-Country Comparisons of Health Facility Readiness to Provide Services (Chapel Hill, NC: Carolina Population Center, University of North Carolina, 2007); and technical consultation with global and local MNH experts.

2.2 Categorizing facilities into five levels

We first divided facilities into three groups based on relatively equal distributions of the facility score: scores from 1 to 5 (no facility had a score of zero); scores of 6 to 9; and scores of 10 to 14. Though the middle group included the smallest range, it included the largest proportion of facilities. Finally, given the vital role that transportation plays in emergency referral, and that the presence of transport is easily

observable and understood by women to affect their chances of being moved quickly to a higher level of care, we moved facilities in the two lowest score groupings (scores of 0 to 9) that had a functioning motor vehicle into their own group. Finally, facilities with the highest scores (between 10 and 14) were moved to the highest group if they had performed cesarean delivery in the previous three months. We considered the recent performance of cesarean delivery as another obvious differentiator likely to influence both women's decisions of where to seek care and providers' decisions of where to refer women. We did not include the performance of blood transfusion, though that is an important service, particularly in the context of hemorrhage; however, readiness to provide blood transfusion was included in the facility score as one of the EmOC signal functions.
