# Supplementary material for: Geographic access to emergency obstetric services: a model incorporating patient bypassing using data from Mozambique
Source: BMJ Glob Health. 2019 Jul 1;4(Suppl 5):e000772. doi: 10.1136/bmjgh-2018-000772 (PMC6606078; doi:10.1136/bmjgh-2018-000772)
Supplement: Supplementary file 3 [file bmjgh-2018-000772supp003.pdf]

Supplementary File 3 for Geographic access to emergency obstetric services: a model incorporating patient bypassing using data from Mozambique

3.1 Building the cost-distance raster layer

Cells representing water features were given null values to exclude them from the model, indicating that pedestrians would not travel across them. The land cover raster was resized to align to the cell size and positioning of the population raster. Additionally, a vector-based river layer was exported from OpenStreetMap[33] converted to a raster, aligned to the population data set, and merged with the land cover raster to ensure that the river feature cells were removed.

Though not every road in Mozambique was found in the OpenStreetMap data set, all primary roads and many secondary roads and paths were included. In areas of the country where the road networks were not yet digitized, especially where a health facility was located, we spent considerable time digitizing additional roads.

Average rates of non-motorized travel across land can be found in Supplementary Table 3.1. We did not increase or decrease these rates of travel for pregnant women because we cannot know the mechanism used to travel along paths to reach a roadway. For example, a woman may have to walk or be carried, which could greatly reduce her rate of travel from the averages below; she could be carried by an animal cart or a motorbike, which would increase the average rate. Once a woman reaches the roadway, our models assumed she will travel by motorized transportation, and there we also used average rates of travel (Supplementary Table 3.2). As with walking paths, the potential modes of transportation are numerous: she could hail a bus that makes multiple stops before reaching the facility; she could hail a taxi that might rush her directly. Therefore, we maintained the standard rates—knowing they are reasonable averages—to minimize assumptions.

Supplementary Table 3.1. Rates of walking across land cover

Classification (EU)	Speed: km/hr	Cost: mins/km
1. Tree Cover, broad-leaved, evergreen	1.000	60
2. Tree Cover, broad-leaved, deciduous, closed	1.000	60
3. Tree Cover, broad-leaved, deciduous, open	1.250	48
4. Tree Cover, needle-leaved, evergreen	1.667	36
5. Tree Cover, needle-leaved, deciduous	1.667	36
6. Tree Cover, mixed leaf type	1.667	36
7. Tree Cover, regularly flooded, fresh water	1.000	60
8. Tree Cover, regularly flooded, saline water	1.000	60
9. Mosaic: Tree Cover/other natural vegetation	1.250	48
10. Tree Cover, burned	1.250	48
11. Shrub Cover, closed-open, evergreen	1.667	36
12. Shrub Cover, closed-open, deciduous	1.667	36
13. Herbaceous Cover, closed-open	1.667	36
14. Sparse herbaceous or sparse shrub cover	2.500	24
15. Shrub and/or Herbaceous Cover, regularly flooded	1.000	60

16. Cultivated and managed	1.667	36
17. Mosaic: Cropland/Tree Cover/other natural veg.	1.667	36
18. Mosaic: Cropland/Shrub and/or Grass Cover	1.667	36
19. Bare	2.500	24
20. Water Bodies (not used - replaced with shipping lane, river, and waterbody layers)	0.000	0
21. Snow and Ice	1.250	48
22. Artificial Surfaces and associated areas	30.000	2

Source: Travel Time to Major Cities: A Global Map of Accessibility. (Europe: European Commission Joint Research Centre, 2015). Available from: http://forobs.jrc.ec.europa.eu/products/gam/sources.php.

Supplementary Table 3.2. Rates of travel along road network

OpenStreetMap Classification	Speed: km/hr	Cost: minutes/ 100 meters
Major roads	60	0.1
Tracks	10	0.6
Major roads (city)	30	0.2
Minor roads	30	0.2

Major roads include primary, primary link, secondary, secondary link, trunk. Major roads (city) include the same types of roads as major but they appear within city limits. Minor roads include roads classified as tertiary, tertiary link, road, unclassified, unnamed. Tracks include footway, pedestrian, path, residential, track, etc.

Source: Travel Time to Major Cities: A Global Map of Accessibility. (Europe: European Commission Joint Research Centre, 2015). Available from: http://forobs.jrc.ec.europa.eu/products/gam/sources.php.
