# Supplementary material for: Geographic access to emergency obstetric services: a model incorporating patient bypassing using data from Mozambique
Source: BMJ Glob Health. 2019 Jul 1;4(Suppl 5):e000772. doi: 10.1136/bmjgh-2018-000772 (PMC6606078; doi:10.1136/bmjgh-2018-000772)
Supplement: Supplementary file 4 [file bmjgh-2018-000772supp004.pdf]

Supplementary File 4 for Geographic access to emergency obstetric services: a model incorporating patient bypassing using data from Mozambique

4.1 Further evidence of the validity of the five-level classification

Level 5 facilities, while accounting for just 5% of facilities overall, saw one-quarter of all institutional deliveries and almost half (48%) of all treated complications (Supplementary Figure 5.1). In Level 4 and Level 3 facilities, utilization was relatively proportional to availability, and was reversed among Level 2 and Level 1 facilities, which had a smaller volume of deliveries and complications relative to the proportion of facilities in those categories.

[Supplementary Figure 4.1]

4.2 Movement across time

We used the modeled scenarios to scrutinize how women would access the various levels of care over time (Supplementary Figure 4.2). In Model 0, within the first hour of travel, 28% of the population would gain access to a Level 5, with the vast majority reaching a Level 5 directly, and just 1% being referred to a Level 5 (after first reaching a lower-level facility). Yet, 69% of the population would remain en route, and an additional 3% would arrive at a lower-level facility and still need to be transferred to a Level 5 in the case of an emergency requiring higher care. By the fifth hour of travel, approximately 61% of the population could arrive at a Level 5 directly, and another 2% could be referred in. At five hours of travel, 21% of the population would not yet have arrived at any facility.

For comparison, Figure 4.2 also presents the same movement across time for Model 3. In this scenario, 29% of the population would directly reach a Level 5 facility within one hour; after two hours of travel, 45% would. At both time points, a very small percentage would be referred into a Level 5 (1% within 1 hour; 1% within 2 hours). Yet, at five hours of travel, 67% of the population would reach a Level 5 directly, and an additional 1% would be referred in from lower-level facilities within five hours. As in Model 0, 21% of the population would remain en route after five hours. Yet, in Model 3, a larger percentage would have arrived at the highest level of care by five hours of travel—68% versus 62% in Model 0, or an additional 1.5 million people (57,000 expected pregnancies and 8,550 expected severe complications).

[Supplementary Figure 4.2]
