# Supplementary figures and images for: Geographic access to emergency obstetric services: a model incorporating patient bypassing using data from Mozambique
Source: BMJ Glob Health. 2019 Jul 1;4(Suppl 5):e000772. doi: 10.1136/bmjgh-2018-000772 (PMC6606078; doi:10.1136/bmjgh-2018-000772)

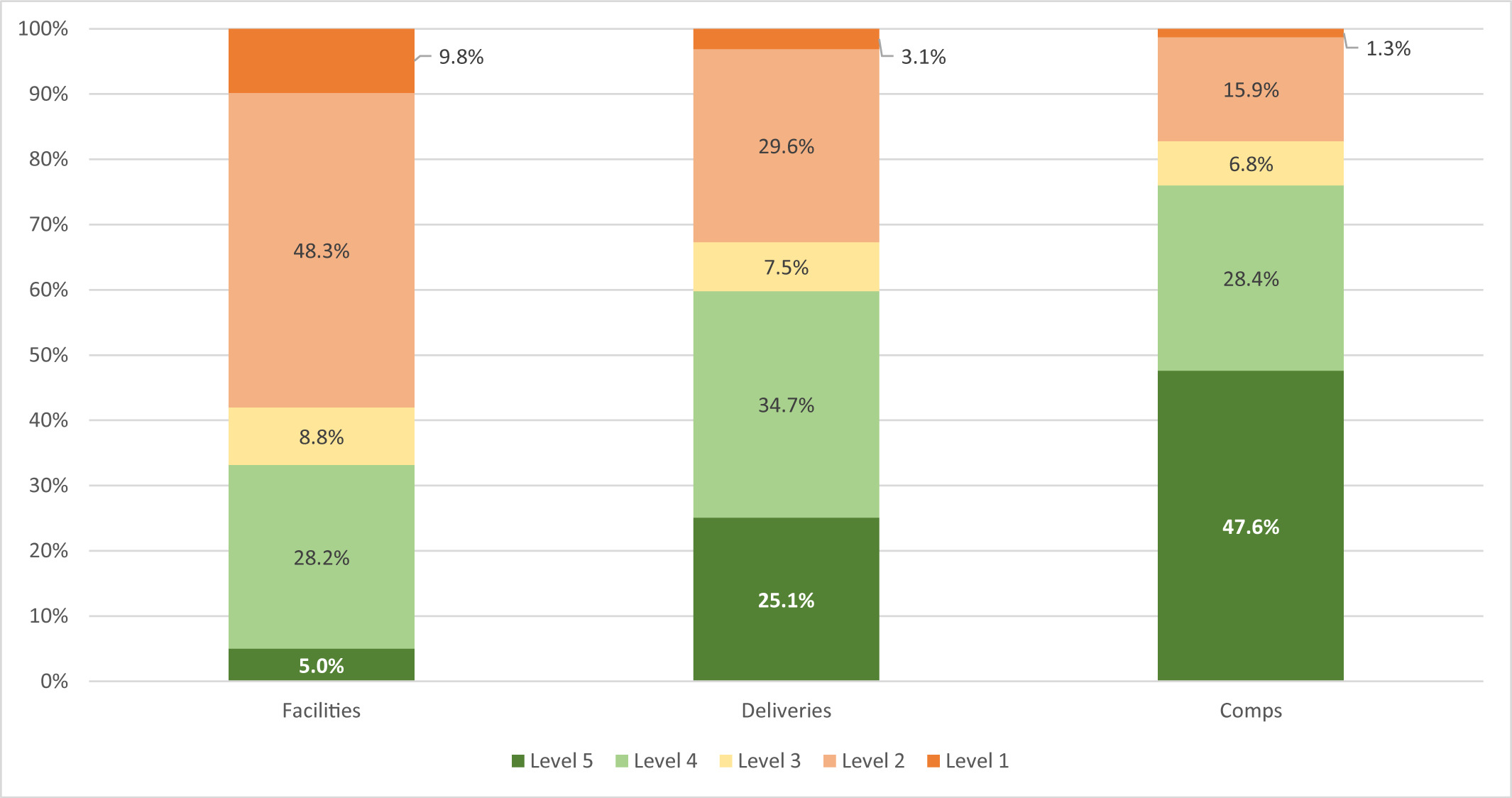

Supplement: Supplementary file 5 [file bmjgh-2018-000772supp005.jpg]

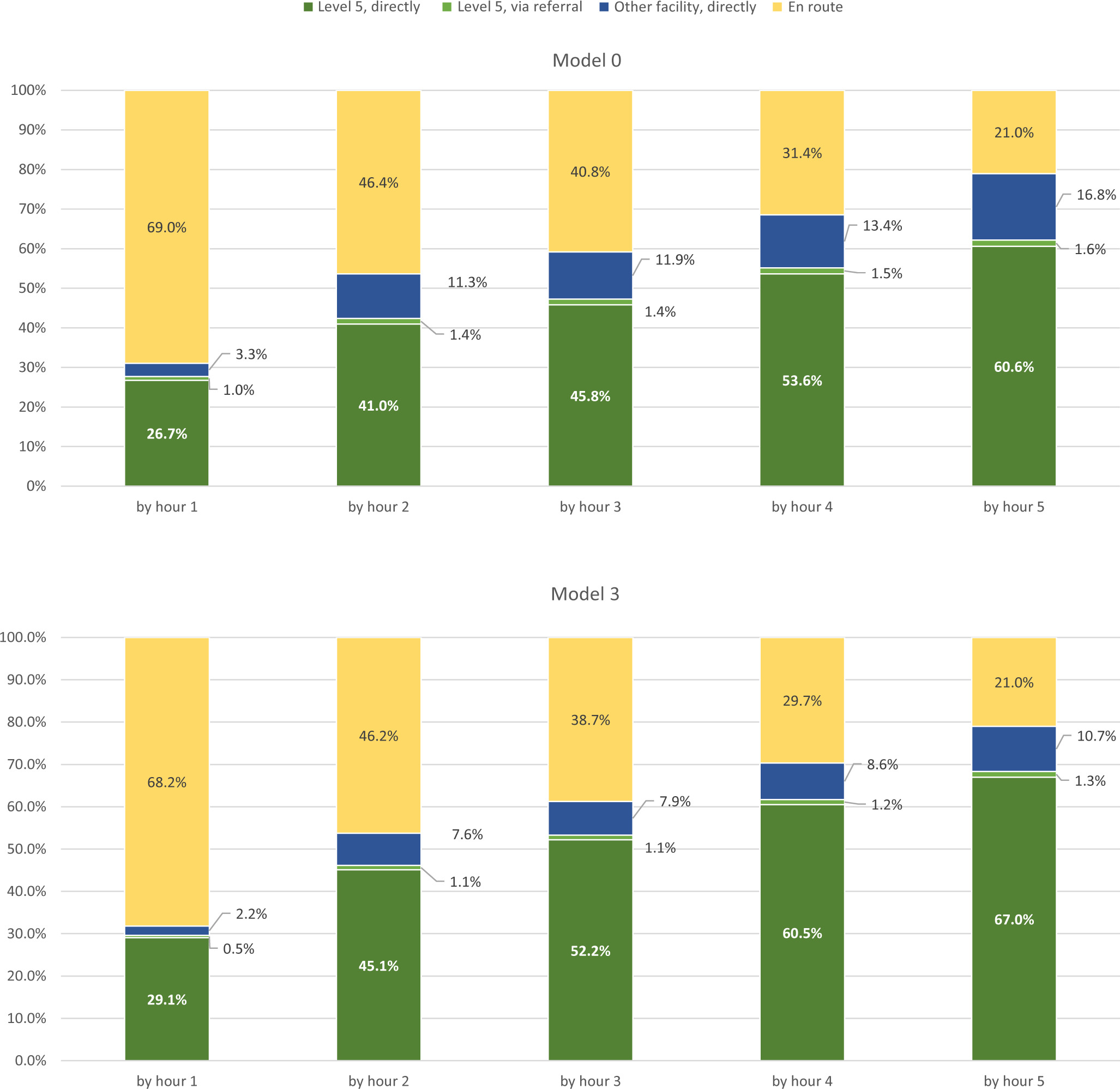

Supplement: Supplementary file 6 [file bmjgh-2018-000772supp006.jpg]
